# Pediatric Research Observing Trends and Exposures in COVID-19 Timelines (PROTECT): Protocol for a Multisite Longitudinal Cohort Study

**DOI:** 10.2196/37929

**Published:** 2022-07-28

**Authors:** Joy Burns, Patrick Rivers, Lindsay B LeClair, Krystal S Jovel, Ramona P Rai, Ashley A Lowe, Laura J Edwards, Sana M Khan, Clare Mathenge, Maria Ferraris, Jennifer L Kuntz, Julie Mayo Lamberte, Kurt T Hegmann, Marilyn J Odean, Hilary McLeland-Wieser, Shawn Beitel, Leah Odame-Bamfo, Natasha Schaefer Solle, Josephine Mak, Andrew L Phillips, Brian E Sokol, James Hollister, Jezahel S Ochoa, Lauren Grant, Matthew S Thiese, Keya B Jacoby, Karen Lutrick, Felipe A Pubillones, Young M Yoo, Danielle Rentz Hunt, Katherine Ellingson, Mark C Berry, Joe K Gerald, Joanna Lopez, Lynn B Gerald, Meredith G Wesley, Karl Krupp, Meghan K Herring, Purnima Madhivanan, Alberto J Caban-Martinez, Harmony L Tyner, Jennifer K Meece, Sarang K Yoon, Ashley L Fowlkes, Allison L Naleway, Lisa Gwynn, Jefferey L Burgess, Mark G Thompson, Lauren EW Olsho, Manjusha Gaglani

**Affiliations:** 1 Abt Associates Atlanta, GA United States; 2 Mel and Enid Zuckerman College of Public Health University of Arizona Tucson, AZ United States; 3 College of Medicine Texas A&M University Temple, TX United States; 4 Leonard M Miller School of Medicine University of Miami Miami, FL United States; 5 Kaiser Permanente Northwest Center for Health Research Portland, OR United States; 6 COVID-19 Response Team Centers for Disease Control and Prevention Atlanta, GA United States; 7 Rocky Mountain Center for Occupational and Environmental Health Department of Family and Preventive Medicine University of Utah Health Salt Lake City, UT United States; 8 St. Luke’s Regional Health Care System Duluth, MN United States; 9 Whiteside Institute for Clinical Research St. Luke’s Duluth, MN United States; 10 Marshfield Clinic Research Institute Marshfield, WI United States; 11 Baylor Scott and White Health Temple, TX United States

**Keywords:** COVID-19, SARS-CoV-2, vaccine effectiveness, vaccine, efficacy, effectiveness, cohort study, pediatric, child, inoculation, vaccination, public health, children, health care professional, health care, caregiver, health data, online survey, incidence

## Abstract

**Background:**

Assessing the real-world effectiveness of COVID-19 vaccines and understanding the incidence and severity of SARS-CoV-2 illness in children are essential to inform policy and guide health care professionals in advising parents and caregivers of children who test positive for SARS-CoV-2.

**Objective:**

This report describes the objectives and methods for conducting the Pediatric Research Observing Trends and Exposures in COVID-19 Timelines (PROTECT) study. PROTECT is a longitudinal prospective pediatric cohort study designed to estimate SARS-CoV-2 incidence and COVID-19 vaccine effectiveness (VE) against infection among children aged 6 months to 17 years, as well as differences in SARS-CoV-2 infection and vaccine response between children and adolescents.

**Methods:**

The PROTECT multisite network was initiated in July 2021, which aims to enroll approximately 2305 children across four US locations and collect data over a 2-year surveillance period. The enrollment target was based on prospective power calculations and accounts for expected attrition and nonresponse. Study sites recruit parents and legal guardians of age-eligible children participating in the existing Arizona Healthcare, Emergency Response, and Other Essential Workers Surveillance (HEROES)-Research on the Epidemiology of SARS-CoV-2 in Essential Response Personnel (RECOVER) network as well as from surrounding communities. Child demographics, medical history, COVID-19 exposure, vaccination history, and parents/legal guardians’ knowledge and attitudes about COVID-19 are collected at baseline and throughout the study. Mid-turbinate nasal specimens are self-collected or collected by parents/legal guardians weekly, regardless of symptoms, for SARS-CoV-2 and influenza testing via reverse transcription-polymerase chain reaction (RT-PCR) assay, and the presence of COVID-like illness (CLI) is reported. Children who test positive for SARS-CoV-2 or influenza, or report CLI are monitored weekly by online surveys to report exposure and medical utilization until no longer ill. Children, with permission of their parents/legal guardians, may elect to contribute blood at enrollment, following SARS-CoV-2 infection, following COVID-19 vaccination, and at the end of the study period. PROTECT uses electronic medical record (EMR) linkages where available, and verifies COVID-19 and influenza vaccinations through EMR or state vaccine registries.

**Results:**

Data collection began in July 2021 and is expected to continue through the spring of 2023. As of April 13, 2022, 2371 children are enrolled in PROTECT. Enrollment is ongoing at all study sites.

**Conclusions:**

As COVID-19 vaccine products are authorized for use in pediatric populations, PROTECT study data will provide real-world estimates of VE in preventing infection. In addition, this prospective cohort provides a unique opportunity to further understand SARS-CoV-2 incidence, clinical course, and key knowledge gaps that may inform public health.

**International Registered Report Identifier (IRRID):**

RR1-10.2196/37929

## Introduction

COVID-19 cases among US children continue to rise, with over 12.7 million reported cases as of March 3, 2022 (representing 19% of all US cases) [1]. Although infection with SARS-CoV-2 typically results in milder symptoms in children than in adults, severe illness resulting in hospitalization, admission to intensive care units, or need for respiratory support can still occur [2-4].

The Pfizer-BioNTech BNT162b2 mRNA COVID-19 vaccine received emergency use authorization (EUA) for individuals aged 16 years and older on December 11, 2020; aged 12-15 years on May 10, 2021; aged 5-11 years on October 29, 2021; and aged 6 months through 5 years June 18, 2022. However, as of March 3, 2022, only 57% and 26% of US adolescents aged 12-17 years and children aged 5-11 years, respectively, are fully vaccinated [5-7].

Randomized controlled clinical trials have shown that the Pfizer-BioNTech COVID-19 vaccine is highly immunogenic and efficacious in preventing infection and severe illness in children and adolescents [8-10]. However, few studies to date have documented vaccine effectiveness (VE) against symptomatic and asymptomatic infection among pediatric populations in real-world conditions. Moreover, sociodemographic and clinical risk factors for SARS-CoV-2 infection in pediatric populations are not well characterized, particularly among those with milder illness; most studies to date have identified risk factors among hospitalized children only [11]. Given the predominance of mild to moderate COVID-19 illness in this age group, assessing the full clinical, immunological, and epidemiological impact of COVID-19 among pediatric populations requires prospective monitoring of both symptomatic and asymptomatic infection.

The HEROES-RECOVER network (Arizona Healthcare, Emergency Response, and Other Essential Workers Surveillance [HEROES] study and the Research on the Epidemiology of SARS-CoV-2 in Essential Response Personnel [RECOVER] study) established a protocol that continues to prospectively collect data on SARS-CoV-2 infections in approximately 6000 health care workers, first responders, and other essential workers [12-14]. Modeled on the HEROES-RECOVER protocols, Pediatric Research Observing Trends and Exposures in COVID-19 Timelines (PROTECT) is a longitudinal prospective pediatric cohort study designed to estimate symptomatic and asymptomatic SARS-CoV-2 incidence and COVID-19 VE against infection among children aged 6 months to 17 years [12,15]. In addition, several secondary objectives are to identify risk factors for SARS-CoV-2 infection and describe the clinical course of COVID-19 in children, such as examining the kinetics of immune responses to SARS-CoV-2 infection. The PROTECT cohort will also be leveraged to monitor influenza infections and vaccine effectiveness, given the similarity in clinical presentation and importance of evaluating influenza vaccines annually.

## Methods

### Study Design

Using a prospective longitudinal cohort design, approximately 2305 children aged 6 months to 17 years will be enrolled, with a planned study duration of at least 15 months after study start. Enrollment began in July 2021 and surveillance is expected to continue through April 2023. Additional study objectives include describing the incidence of SARS-CoV-2 infections by age, sociodemographic characteristics, health status, and other risk factors. Primary and secondary study objectives can be found in Textbox 1.

**Primary and secondary objectives of the PROTECT (Pediatric Research Observing Trends and Exposures in COVID-19 Timelines) study.**

*Primary Objectives*

- Contribute to estimates of vaccine effectiveness (VE) of authorized COVID-19 vaccines in preventing laboratory-confirmed symptomatic and asymptomatic SARS-CoV-2 virus infections among children aged 6 months to 17 years.
- Contribute to estimates of the incidence of laboratory-confirmed SARS-CoV-2 infection, including asymptomatic and symptomatic infections, using molecular, virologic, and serologic diagnostics, and describe differences by age and other sociodemographic characteristics, health status, and/or other risk factors.

*Secondary Objectives*

- Contribute data to explore the VE, impact, and uptake of authorized COVID-19 vaccines.
- Describe associations between COVID-19 vaccination and SARS-CoV-2 infection and symptoms by vaccine product, number of doses, and timing of vaccination.
- Describe VE by age and other sociodemographic characteristics, health status, and/or other risk factors.
- Examine the association between vaccination and illness severity, duration, and infectiousness (or viral shedding) among children with SARS-CoV-2 infection.
- Describe child-reported adverse reactions following vaccination.
- Contribute data to explore clinical and epidemiological factors of SARS-CoV-2 infection.
- Examine the kinetics of immune responses to SARS-CoV-2 infection.
- Describe the association between preexisting SARS-CoV-2 antibodies and subsequent risk of SARS-CoV-2 reinfection.
- Describe the clinical characteristics and functional impact associated with COVID-19 illness among children.
- Contribute data to characterize knowledge, attitudes, and practices of parents/guardians related to COVID-19 vaccines, and examine associations with vaccine hesitancy and uptake or adherence to vaccination recommendations.
- Contribute data to examine the duration of viral RNA detection associated with symptomatic and asymptomatic SARS-CoV-2 infection using quantitative molecular methods.
- Contribute data to examine the incidence and outcomes of other respiratory virus infections, including influenza virus infection.
- Contribute to VE estimates of influenza vaccines in preventing influenza virus infection and influenza illness.

### Setting

PROTECT is an ancillary study to the HEROES-RECOVER network, which includes prospective cohorts from two studies: the HEROES study and the RECOVER study [12,15]. The RECOVER sites encompass five US states: Florida (Miami), Minnesota (Duluth), Oregon (Portland), Texas (Temple), and Utah (Salt Lake City). The HEROES study is based in Arizona (Phoenix, Tucson, and other areas). Study teams from the Arizona, Florida, Texas, and Utah sites are recruiting children into the PROTECT study, and principal investigators from all sites, including the Minnesota and Oregon sites, provided input on study design. PROTECT recruits children from households within the HEROES-RECOVER cohort as well as from the surrounding community. The Minnesota and Oregon sites do not recruit participants for PROTECT.

### Eligibility Criteria

Eligible children include children aged 4 months to 17 years at the date of recruitment with plans to stay in the area for the next 12 months, although children 4-5 months old do not enroll and start study activities until they are 6 months old. If a child turns 18 years of age during the study period, they are either reconsented or have consent waived depending on site institutional review board (IRB) requirements and can continue to participate.

The child’s parent or legal guardian must have access to a smartphone or internet-connected computer, a mailing address where they can receive study supplies, willingness and ability to complete periodic data collection activities, and the ability to speak and understand English or Spanish (they must sign the English- or Spanish-language study consent form).

Exclusion criteria include receiving both doses of a two-dose COVID-19 vaccine (or one dose of a single-dose COVID-19 vaccine if authorized during the study period) ≥14 days prior to enrollment (for those enrolled at the Texas, Utah, and Florida study sites) or participating in a vaccine trial within 3 months of the enrollment date. To ensure representation of both vaccinated and unvaccinated children, children recruited from the community are required to be either unvaccinated or only partially vaccinated. The HEROES study site (Arizona) used a target ratio of 1:6 participants vaccinated against COVID-19 to unvaccinated participants to direct the recruitment strategy. Some recruitment for the pediatric cohort occurred prior to vaccine availability for those under 12 years of age (see Table 1).

**Table 1:** PROTECT (Pediatric Research Observing Trends and Exposures in COVID-19 Timelines) study sites and site characteristics.

Characteristics	University of Arizona	Baylor Scott & White Health	University of Miami	University of Utah
Location	Tucson, Arizona	Temple, Texas	Miami, Florida	Salt Lake City, Utah
Recruitment area	Entire state of Arizona	Bell, Burnet, Coryell, Falls, Lampasas, McLennan, Milam, and Williamson counties in Texas	Miami-Dade county	Salt Lake, Davis, Utah, Summit, Morgan, Weber, and Tooele counties
Population of children <18 years of age in the recruitment area^a^ [16]	1,609,000	363,000	546,000	758,000
Recruitment goal	1500	200	330	275
Recruitment strategies	Websites, social media, press releases, outreach to community members and interested parties, current HEROES^b^ households	Radio, websites, search engines, social media, targeted phone banking, outreach to community members and interested parties, current RECOVER^c^ households	Radio, websites, search engines, social media, targeted phone banking, outreach to community members and interested parties, current RECOVER households, address-based sampling mailers	Radio, websites, search engines, social media, outreach to community members and interested parties, current RECOVER households
Eligibility related to vaccination status^d^	Varied based upon vaccine availability at the time of enrollment	Not fully vaccinated at enrollment unless parents/legal guardians participate in RECOVER	Not fully vaccinated at enrollment unless parents/legal guardians participate in RECOVER	Not fully vaccinated at enrollment unless parents/legal guardians participate in RECOVER

^a^Rounded to the nearest thousand. Data from April 2020.

^b^HEROES: Healthcare, Emergency Response, and Other Essential Workers Surveillance study.

^c^RECOVER: Research on the Epidemiology of SARS-CoV-2 in Essential Response Personnel study.

^d^See Table 3 for definitions of vaccination status.

### Recruitment Strategy

The PROTECT study’s enrollment goal of 2305 children is intended to assure adequate statistical power (see detailed power calculations below) after accounting for nonresponse and attrition. Sites enroll children across three age groups: children aged 6 months to 6 years, aged 7-11 years, and aged 12-17 years. Each site has recruitment goals based on its catchment area (see Table 2). Site-specific enrollment targets are reflective of the current enrollment totals of the parallel cohort study among frontline workers from which children would be recruited. Sites were asked to project an additional number of participants that could be recruited from community engagements of the sites. Study sites implement a multipronged recruitment strategy, including: (1) enrolling from the existing HEROES-RECOVER cohort; (2) sharing study information with health care systems and providers, community organizations, school districts, and vaccination sites; and (3) accepting self-referrals from community marketing tailored to the site, which may include press releases, social media, radio ads, and Google banners.

**Table 2:** PROTECT (Pediatric Research Observing Trends and Exposures in COVID-19 Timelines) enrollment goal by study site and age group.

Age group	Arizona HEROES^a^	Texas	Florida	Utah	All PROTECT
6 months to 6 years	300	63	70	105	538
7-11 years	700	63	105	105	973
12-17 years	700	74	155	40	969
Total	1500	200	330	Up to 275	2305

^a^HEROES: Healthcare, Emergency Response, and Other Essential Workers Surveillance study.

Recruitment and enrollment activities vary by site but may be conducted by a telephone call with study staff, a virtual call center, or in-person at study site clinics. Participants’ parents/legal guardians may opt to complete a screening interview during recruitment or via the study website to determine eligibility and interest. Study staff inform parents/legal guardians of the participation incentives, including gift cards or Clin cards (reloadable prepaid gift cards) for completion of study activities, quarterly gift cards, or raffles, varying by site and local IRB guidance (see Table S1 in Multimedia Appendix 1).

### Data Collection

Data are collected and managed through REDCap (Research Electronic Data Capture), a secure, online Health Insurance Portability and Accountability Act–compliant data management platform, which is only accessible through a password-protected REDCap account [17,18]. Sites use Mosio or Twilio to automate the process of sending regular text messages or emails containing specimen collection reminders and links to the secure data collection platform [19,20]. For the Utah and Texas study sites, data are stored through a centralized REDCap project managed by Abt Associates and housed at Vanderbilt University. The Florida and Arizona study sites maintain their own REDCap databases through their universities.

All study-related documents and samples contain a unique identifier for each child. Additionally, a unique identifier is created at the household level, given that multiple children from a household may enroll in PROTECT. Study data are linked with this household identifier to help facilitate the distribution of study supplies to parents/legal guardians and allow for clustering analysis.

### Enrollment

An electronic copy of the study consent and assent forms is sent to parents/legal guardians for review. Study staff review all information and study procedures with the parents/legal guardians. Study staff document written permission from a parent/legal guardian for the child to participate in the study, allow study staff to store biospecimens (nasal specimens and blood samples) after PROTECT ends, and obtain written consent for their participation in study survey data collection. Study staff also obtain and document written assent from children aged 12-17 years or verbal assent from children aged 7-11 years prior to starting enrollment activities. The parent/legal guardian must provide permission for each eligible child if multiple children from their household participate in PROTECT.

After consent, an enrollment survey for each child enrolled in PROTECT is completed by the parent/legal guardian to record information on household composition, household members’ COVID-19 vaccination status, perceptions of COVID-19 and COVID-19 vaccines (and intent if the child is not vaccinated), COVID-19 illness history, sociodemographic information (such as household income, race/ethnicity, and current gender identity), school and/or daycare attendance, participation in extracurricular and work-related activities, health insurance coverage, percent of time masks are worn in school/daycare and the community, medical history (eg, influenza and other routine childhood vaccines, medical conditions, daily medication use, and any hospitalizations in the past 12 months), and self-reported overall health status and sleep quality (see Table S2 in Multimedia Appendix 2).

Parents/legal guardians are provided with multiple respiratory specimen collection kits per enrolled child. These kits include study information and all specimen collection materials. Specimen collection kits are replenished periodically to ensure participants do not run out. Parents/legal guardians are given written and visual detailed instructions for collecting, storing, and submitting specimens. Depending on the study site, parents/legal guardians may drop specimens off at a specified location, have them picked up by an at-home courier, or ship them according to FDA guidance and specifications.

The parent/legal guardian also selects which day of the week they agree to collect and return the kit. They will do this on the same day every week, regardless of whether the child is experiencing illness symptoms or not. The evening prior to collecting the child’s nasal specimen, parents/legal guardians may receive a reminder email or text message, depending on the study site.

### Active Surveillance

Each week, children enrolled in PROTECT provide a mid-turbinate nasal specimen and the parent/legal guardian responds to three questions in writing on the specimen collection bag: the collection date of the respiratory specimen, whether the child is experiencing COVID-like illness (CLI) symptoms, and how many days ago CLI symptoms began (if appropriate). For PROTECT, CLI is defined as the presence of at least one of the following symptoms: fever, chills, cough, shortness of breath, sore throat, diarrhea, muscle or body aches, and change in smell or taste. For nonverbal children, the following symptoms are also included: runny nose, fatigue/being run-down, decreased activity, and irritability/crankiness.

A parent/legal guardian collects the nasal specimen using a flocked nasal swab and places it in a viral transport medium (VTM) for children aged 6 months to 11 years. For children aged 12-17 years, the child has the option to self-collect the nasal specimen or a parent/legal guardian can collect it for them. Parent/legal guardian responses on the collection bag and the results of the reverse transcription-polymerase chain reaction (RT-PCR) tests drive potential survey pathways as described below.

**Figure 1:**
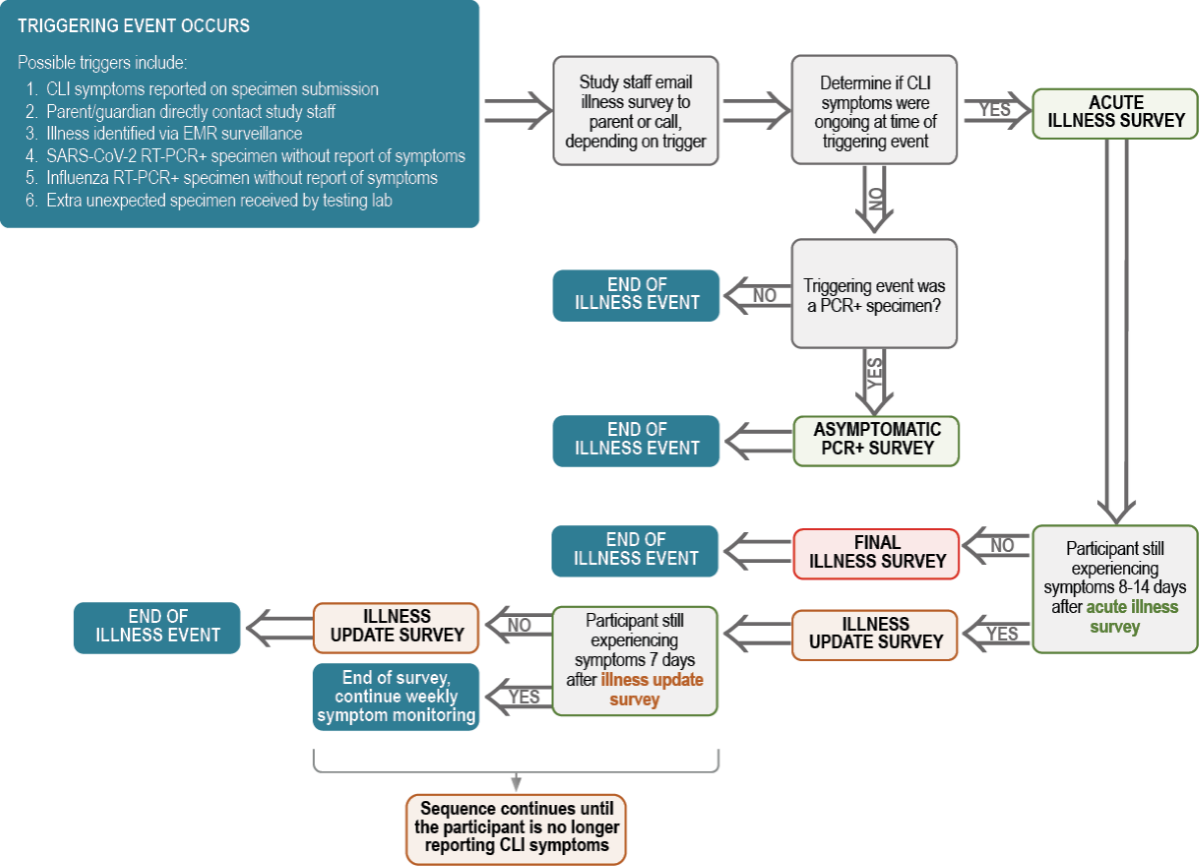
PROTECT active surveillance flowchart. EMR: electronic medical record; CLI: COVID-like illness; RT-PCR: reverse transcription-polymerase chain reaction.

A positive RT-PCR result triggers several study-related activities, starting with reporting the result to the parent/legal guardian and state/local registries. Study staff begin an illness survey (described in Figure 1) and offer parents/legal guardians the opportunity for the participant to provide a COVID-19 convalescent blood draw. Additionally, the study staff ensures that the participant’s vaccine information is up to date in REDCap by reminding parents/legal guardians to complete the vaccine update survey and/or accessing vaccine data within electronic medical records (EMRs) or state registries.

If a specimen has a positive RT-PCR test result for SARS-CoV-2 or influenza A/B, or CLI symptoms are reported on the specimen collection bag, the study staff receives an email alert from REDCap. Likewise, parents/legal guardians may also report a positive COVID-19 test result obtained from outside PROTECT to study staff. If a child is experiencing any CLI symptoms according to the study’s case definition on a day other than their predetermined collection day, the parent/legal guardian is directed to collect and submit an additional nasal specimen. Where study sites can access the EMR for research, study staff may also identify acute illness through daily monitoring of medical visits for an acute respiratory illness.

After either a CLI or RT-PCR–positive test result is noted for the child, study staff send the parent/legal guardian an illness survey to complete or the survey is completed with the parent/legal guardian over the phone. In the survey, parents/legal guardians are asked to describe the child’s symptoms, subjective illness duration and severity (including missed school/daycare, ability to do normal activities, and days spent in bed), medical utilization, impact on school/daycare/work/extracurricular attendance, and potential exposures. In addition, if the participant is RT-PCR–positive for influenza, parents/legal guardians are asked whether the child has received the flu nasal vaccine, because positive influenza results can occur after recent administration of such vaccines [21,22]. If the child has an RT-PCR–positive result for influenza only, the parent/legal guardian is not asked about direct contact with individuals with COVID-19 at daycare/school or in the community. If the parent/legal guardian reports that the child continues to experience symptoms 7 days after the initial illness survey, they are asked to complete a one-time illness update survey to assess new symptoms, subjective illness severity (including missed school/daycare, ability to do normal activities, and days spent in bed), and medical utilization.

Once symptoms are no longer reported, the parent/legal guardian is asked to complete a final illness survey to assess symptoms experienced throughout the duration of the illness, subjective illness severity (including missed school/daycare, ability to do normal activities, and days spent in bed), whether the child has received the flu nasal vaccine (if RT-PCR–positive for influenza), and whether they used medical care over the course of the entire illness. At this point, the illness event is considered over. If new or additional CLI symptoms are reported (or the child receives a new positive RT-PCR result), a new illness event begins and the parent/legal guardian receives a new series of illness surveys to complete.

This process also occurs if the child experiences a new positive RT-PCR result and does not report CLI symptoms; the parent/legal guardian completes an asymptomatic RT-PCR–positive survey online or over the phone with study staff. This survey, like the acute illness survey, asks the parent/legal guardian if the child has experienced any non-CLI symptoms. If they report symptoms, they describe subjective illness severity, duration, medical utilization, impact on school/daycare/work/extracurricular attendance, potential exposures, and whether the child has received the flu nasal vaccine (if RT-PCR–positive for influenza). If the child has an RT-PCR–positive result for influenza only, the parent/legal guardian is not asked about direct contact with individuals with COVID-19 at daycare/school or in the community.

### Monthly and Quarterly Surveys

In addition to the weekly active surveillance, parents/legal guardians receive a monthly survey that asks them to provide updated information about their participating child’s vaccination status; any COVID-19 vaccine–related side effects; in-person school or daycare attendance; participation in extracurricular, work-related, and social activities; and mask-wearing habits. Quarterly, this survey also asks about their participating child’s health status; illnesses not reported in the previous 3 months; the parent/legal guardian’s knowledge, attitudes, and perceptions of COVID-19 and COVID-19 vaccination; and any other childhood vaccines received. Parents/legal guardians receive a link to these surveys via email or text message and complete them in REDCap.

### Electronic Medical Records

At the Texas site, data from EMRs are abstracted to count medical visits for acute COVID-19 illness, identify influenza and COVID-19 vaccinations, and assess chronic medical conditions for the 12 months before enrollment and through the end of the study. The EMR extraction uses the International Statistical Classification of Diseases and Related Health Problems, 10th revision codes for all ambulatory medical encounters and all hospital admissions.

### COVID-19 Vaccine Information

COVID-19 vaccination information is captured in several ways: via data abstracted and extracted from the child’s EMR and/or state or municipal vaccine registry, as well as based on parent/legal guardian report. Using multiple methods to collect this information ensures complete and near real-time data capture, which is a critical component of monitoring for receipt of additional doses and allows study staff to cross-check vaccination information received from multiple sources to ensure accuracy. The use of multiple data sources also enables more accurate designation of the child’s current vaccination status, particularly for unvaccinated and partially vaccinated children, by ensuring that the study team can correctly track and appropriately designate a change in vaccination status in a timely manner. No COVID-19 vaccines are offered to participants as part of enrolling in PROTECT. The definitions describing a child’s COVID-19 vaccination status are provided in Table 3.

All parents/legal guardians of children who are eligible for a COVID-19 vaccine—and do not have vaccination documented from a state or municipal vaccine registry—receive a COVID-19 vaccination update survey asking about the child’s COVID-19 vaccination status (as described in Multimedia Appendix 2). If the parent/legal guardian reports that the child has not been vaccinated against COVID-19, they are asked if they expect that the child will receive a vaccine in the next 8 weeks, and, if so, in how many weeks. This timeline is used to inform when the follow-up COVID-19 vaccination update survey is sent; if the parent/legal guardian reports the number of weeks until vaccination, the survey is sent at that time. Otherwise, a follow-up COVID-19 vaccination update survey is sent in 8 weeks. Follow-up COVID-19 vaccination update surveys are sent on this schedule until the parent/legal guardian reports that the child is fully vaccinated against COVID-19.

If the parent/legal guardian reports that the child has been vaccinated, they are asked the child’s date of vaccination, number of doses received, vaccine manufacturer, and location of vaccination. They are also asked to upload a copy of the child’s vaccine card for study staff to verify the information.

If a child is fully vaccinated and eligible to receive a booster vaccine, the parent/legal guardian is sent a booster survey every 12 weeks as long as the child is eligible to receive a booster vaccine. If the parent/legal guardian reports that the child has received a booster vaccine, they are asked the date of vaccination, vaccine manufacturer, and location of vaccination.

**Table 3:** COVID-19 vaccination status definitions.

Term	Description
Unvaccinated	No COVID-19 vaccine doses received
Partially vaccinated	≥14 days after the first dose in the primary series
Fully vaccinated	≥14 days after final dose in the primary series of a multidose vaccine or one dose of a single-dose vaccine, if authorized during the study period^a^
Boosted	≥7 days after any booster/additional dose
Indeterminate	<14 days after the first dose in the primary series

^a^At the time of publication, current COVID-19 vaccine recommendations include a primary series and booster doses that vary by age and vaccine product. Currently, Pfizer-BioNTech BNT162b2 primary vaccine series includes 2 doses for children ages 5-17 years and 3 doses for children 6 months to 5 years; a booster dose is recommended at least 5 months after the final dose in the primary series for children aged 12-17 years. Moderna mRNA-1273 primary series includes 2 doses for children 6 months to 17 years; at this time, a booster is not recommended for pediatric Moderna recipients.

### Laboratory Methods

#### Respiratory Specimens

Each week, children enrolled in PROTECT provide a mid-turbinate nasal specimen collected using a flocked nasal swab, which is placed in VTM and shipped with a cold pack using priority overnight shipping (all specimen collection materials are FDA-approved or authorized for SARS-CoV-2 and influenza RT-PCR testing). Respiratory specimens are sent to Marshfield Clinic Research Laboratories in Marshfield, Wisconsin, for RT-PCR testing for SARS-CoV-2 and influenza A/B using the Thermo Fisher TaqPath COVID-19, FluA, FluB Combo Kit assay, which is authorized for emergency use by the FDA [23]. Testing for influenza is conducted only during periods of relevant viral circulation and is completed using protocols, primers, probes, and reagents approved by the Centers for Disease Control and Prevention (CDC). Test results are returned to study sites via REDCap so they may notify parents/legal guardians of the child’s results and flag RT-PCR–positive specimens for follow-up. Each study site complies with the reporting requirements of their state and local public health departments. The remaining aliquots of all study specimens are sent to a CDC-designated facility for additional virus characterization (including but not limited to genetic sequencing and novel severity markers), banking, and storage.

#### Blood Specimens

Blood draws are not required for participation in the PROTECT study. Participating children, with their parent/legal guardian’s permission, may opt into a blood draw at each of the following opportunities: at enrollment, following SARS-CoV-2 infection (if any), following completion of a COVID-19 primary vaccine series, and following each booster dose. They may decline any blood draw at any time. Although at least 5-10 mL of whole blood is preferred, 3 mL may be accepted from infants and young children, with up to 20 mL collected from older children, depending on the study site. In the event of a SARS-CoV-2 infection, the blood sample is requested approximately 4 weeks after illness onset or RT-PCR detection if the child does not develop symptoms. The postvaccination sample is requested 10-60 days after completion of a COVID-19 vaccine primary series and each booster dose (see Table 3 for definitions).

Whole blood is collected and processed by the study site laboratory using CDC guidelines for serum collection [24]. Serum specimens are tested with CDC-approved serologic assays. The CDC laboratory or a CDC-specified reference laboratory will conduct serologic work and report results to the CDC investigators. After testing, any leftover serum will be stored for possible additional testing in the future.

### Statistical Considerations

#### Data Quality

Branching logic, date validation, range checks, and automated skip patterns are used throughout the REDCap project to ensure high-quality data collection. Additionally, study staff perform weekly quality checks for out-of-range values and missing or inconsistent data. Sites review the results of these quality checks and follow up with the parent/legal guardian for verification or correction if necessary.

#### Power Analysis for VE

Recruitment goals for the minimum necessary sample size were established using Monte Carlo simulation methods that account for time-varying vaccination status, amount of observed person-time at risk for infection in both the vaccinated and unvaccinated groups, the SARS- CoV-2 incidence rate (the “background” rate of infection), and the true underlying effect (VE) size. A time-dependent Cox proportional hazards model was fit to estimate with a hypothetical cohort of 1780 and 9 months of surveillance. Robust standard errors were used to account for the clustering by study location [25]. The simulation accounted for the distribution of participants by site and age group, assumed 15% attrition, and site- and age-specific estimates of SARS-CoV-2 monthly attack rate ranging from 0.25% to 1.2%. The calculation found that the study will have sufficient power to detect at least one breakthrough SARS-CoV-2 infection and calculate VE with a lower confidence interval bound >0. Recruitment/enrollment goals were set higher than this ongoing target sample size to account for expected attrition. Although the power simulation accounted for attrition, an additional count of participants was added to provide the study with the capacity to address unforeseen changes and needs given the pandemic environment. Table 2 illustrates the recruitment goal by site by age.

Depending on the sample size, VE may be calculated by age group, full versus partial vaccination status, by booster or additional dose status, and vaccine type, if multiple products become available to use. The unadjusted incidence of symptomatic or asymptomatic SARS-CoV-2 infections will be calculated as the number of cases in the cohort over the number of person-weeks contributed by children and their associated 95% CIs. 

### Statistical Analysis

#### Vaccine Effectiveness

COVID-19 VE among participating children will be estimated using the Andersen-Gill extension of the Cox proportional hazards model. In this model, person-time would be counted according to the times indicated in Table 2 until the end of the follow-up period or SARS-CoV-2 detection. Unadjusted VE will be calculated as 100%×(1–hazard ratio for SARS-CoV-2 infection in vaccinated vs unvaccinated children). An adjusted model will utilize an inverse probability of treatment weighting approach with individual propensities to be vaccinated in each week based on associated characteristics such as sociodemographic characteristics, health information, exposure variables, mask usage, and local viral circulation. These predicted propensities will be used to calculate stabilized weights that are incorporated into a Cox proportional-hazards model.

#### Incidence

The unadjusted incidence of symptomatic or asymptomatic SARS-CoV-2 infections will be calculated as the number of cases in the cohort over the number of person-weeks contributed by participants and 95% CIs, assuming a binomial distribution. The adjusted incidence will be calculated using a negative binomial model, adjusted for age, sex, race, ethnicity, and other potential confounders. The incidence of other respiratory viruses will be calculated as laboratory-confirmed infection data are available.

### Ethical Considerations

This study protocol was reviewed and approved by the Abt Associates IRB (which serves as the single IRB of record for the Florida, Texas, and Utah sites and the CDC; approval number 2109), by the University of Arizona IRB for the Arizona site, and the CDC per the US Department of Health and Human Services Policy for Protection of Human Subjects (45 C.F.R. part 46). All parents/legal guardians complete informed consent, with children completing written or verbal assent as appropriate. Study staff verifies that parents/legal guardians, and children, understand the study activities and objectives and are aware of any associated risks before enrollment and study participation.

## Results

Enrollment began on July 27, 2021; as of May 13, 2022, 2371 children are enrolled in the PROTECT study, 20% of whom are aged 6 months to 4 years, 59% aged 5-11 years, 16% aged 12-15 years, and 5% aged 16-17 years. Enrollment will continue until the study site targets are met or exceeded, with data collection planned to continue through at least October 2022. This age breakdown reflects COVID-19 vaccine EUA age groups. In December 2021, PROTECT published interim VE estimates among adolescents aged 12-17 years [26].

## Discussion

### Projected Significance

The PROTECT cohort will provide critical information that allows estimation of SARS-CoV-2 incidence and COVID-19 VE in the real world among children and adolescents with relevance to inform public health policy. PROTECT will prospectively examine VE of authorized COVID-19 vaccines in children aged 6 months to 17 years in real-world conditions, relying on prospective monitoring to capture both symptomatic and asymptomatic SARS-CoV-2 infection. Active surveillance and follow-up are of particular importance for children, who are more likely than adults to experience mild or asymptomatic infections [27].

The study will additionally describe SARS-CoV-2 incidence, severity, and risk factors in children and adolescents, including those with mild, moderate, or no symptoms. While risk factors in pediatric patients are not well characterized, differences in adults by sociodemographic characteristics have been previously established [28,29]. This prospective cohort study addresses this need, while also providing data to estimate and further describe CLI in children and assess the extent of coinfections such as respiratory syncytial virus and influenza.

It is particularly important to understand these factors in the present context of US children’s increased exposure to SARS-CoV-2 infection from in-person K-12 schools and daycare settings, especially in the wake of the recent surge in cases due to the emergence of the Omicron variant. The pandemic and responses to it have disrupted US children’s education and development [30,31]. Information on sociodemographic and medical characteristics associated with COVID-19 in the pediatric population may influence policy decisions around school closures, quarantine periods, mask mandates, and other preventive measures.

Information gathered from the serial blood sampling component of the study will allow for examinations of immune response to infection and vaccination in children and the presence of antibodies from prior infections. Differences found in immune responses from infection and/or vaccination, as well as analysis of antibody longevity and potential waning protection can help to inform decisions around vaccine recommendations and the booster schedule.

### Strengths

PROTECT provides a data-driven platform for contributing to CDC assessments of multiple SARS-CoV-2 and influenza research objectives in a pediatric population across multiple age groups and sociodemographic characteristics in multiple geographic settings. Moreover, PROTECT’s verification of vaccines with state registries, and EMR where available, promote data quality.

The multisite cohort design can account for the heterogeneity of SARS-CoV-2 incidence as well as other factors related to geographic variability. Another strength is the inclusion of children and adolescents with potentially high exposure to SARS-CoV-2 through school, daycare, extracurricular activities, and the community. The longitudinal cohort study design allows for continuous and consistent assessment of symptoms, CLI, exposure, and SARS-CoV-2 infection and VE. Depending on sample size, VE may be calculated by age group, full versus partial vaccination status, by booster or additional dose status, and vaccine type, if multiple products become available to use. Finally, because one of the primary recruitment pools is the existing HEROES-RECOVER network, a high percentage of children come from households with health care personnel, first responders, and other essential frontline workers, making this study an important contributor to understanding COVID-19 VE and incidence in children of workers who are at risk for high exposure.

### Limitations

This study is subject to several limitations. First, the ability to generalize trends in SARS-CoV-2 infection, disease severity, and immunological response in children may be biased based on enrollment, as those who participate in this type of study may be more likely to immunize their children. Moreover, PROTECT is recruiting in part directly from the HEROES-RECOVER network, which is likely biased by the healthy worker effect [32]. Adults who choose to participate in the HEROES-RECOVER network are required to meet a baseline level of health (eg, physical examinations to qualify for employment as first responders) or have specialized health care knowledge for their professions, which may potentially lead to differences in knowledge, attitudes, and practices around vaccinating children in their household against COVID-19 that differ from those of the general population. Second, the information received from children is self-reported, which can introduce error if the parents/legal guardians of children are not able to provide consistent information related to specimen collection, COVID-19, or influenza vaccination, and potential CLI via illness surveys. Third, there may be selection bias due to the requirement of phone and internet access to complete surveys and the requirement for children to reside at a permanent address. Fourth, nasal specimens are collected by parents/legal guardians or participants, and therefore may have a higher rate of error than if the specimens were collected by trained health care professionals. Additionally, because the PROTECT cohort includes select study sites in multiple states and is not nationally representative, the generalizability of study findings and results may be limited. Further limitations of PROTECT include relatively low enrollment and difficulty of participants adhering to the study activities. Because of experience with the HEROES-RECOVER adult cohorts, the study team predicted that adherence and retention were likely challenges. Currently, adherence of weekly nasal specimens is approximately 85%, and a total of 169 participants have withdrawn.

### Conclusions

The study design, recruitment strategies, research activities, and pediatric population of the PROTECT study create a unique opportunity to further understand SARS-CoV-2 infection, illness, and VE in children and adolescents residing in multiple geographic areas throughout the United States. Ongoing monitoring of symptoms may allow researchers to further refine the characterization of CLI in children. This study also examines the knowledge, attitudes, and practices of parents as they relate to the COVID-19 vaccines in children and adolescents. Finally, the study is designed to explore possible risk factors associated with both symptomatic and asymptomatic illness and disease severity in both vaccinated and unvaccinated children, which are of interest to public health, child development, and education policymakers.

## Appendix

Multimedia Appendix 1 Incentives for PROTECT (Pediatric Research Observing Trends and Exposures in COVID-19 Timelines).

Multimedia Appendix 2 PROTECT (Pediatric Research Observing Trends and Exposures in COVID-19 Timelines) study data collection activities.

## Abbreviations

CDC: Centers for Disease Control and Prevention
CLI: COVID-like illness
EMR: electronic medical record
EUA: emergency use authorization
FDA: Food and Drug Administration
HEROES: Healthcare, Emergency Response, and Other Essential Workers Surveillance
IRB: Institutional Review Board
PROTECT: Pediatric Research Observing Trends and Exposures in COVID-19 Timelines
RECOVER: Research on the Epidemiology of SARS-CoV-2 in Essential Response Personnel
REDCap: Research Electronic Data Capture
RT-PCR: reverse transcription-polymerase chain reaction
VE: vaccine effectiveness
VTM: viral transport medium
