# Supplementary material for: Pediatric Research Observing Trends and Exposures in COVID-19 Timelines (PROTECT): Protocol for a Multisite Longitudinal Cohort Study
Source: JMIR Res Protoc. 2022 Jul 28;11(7):e37929. doi: 10.2196/37929 (PMC9377426; doi:10.2196/37929)
Supplement: Multimedia Appendix 1 [file resprot_v11i7e37929_app1.docx]

**Table S1. Incentives for PROTECT (Pediatric Research Observing Trends and Exposures in COVID-19 Timelines)**

|  | Enrollment goal, incentives | Description | Cost per item | Incentive distribution |
| --- | --- | --- | --- | --- |
| University of Arizona | 1500 |  |  |  |
| **Incentives** | |  |  |  |
|  | Incentive 1 | Blood draw for serum (up to 20 mL) | $50 |  |
|  | Incentive 2 | Weekly mid-turbinate nasal swab (and illness survey if applicable) | $15 | Per University policy, incentives are distributed to PLGs via Paypal, Venmo, or ClinCard (or to participants who have turned 18 while in the study) |
| BSWH | 200 |  |  |  |
| **Incentives** | |  |  |  |
|  | Incentive 1 | $40 for enrollment survey completion and follow-up instructions, $20 per blood draw, and $10 per mid-turbinate swab collection and return | $40 for enrollment survey completion and follow-up instructions, $20 per blood draw, and $10 per mid-turbinate swab collection and return | BSWH will issue gift cards. Parents/guardians who are Baylor Scott and White employees have the option to receive payment in their paychecks as well. |
|  | Incentive 2 | Enrollment survey and follow up instructions | $40 |  |
|  | Incentive 3 | Blood draw for serum (3-5 mL) | $20 |  |
|  | Incentive 4 | Mid-turbinate swab collection and shipping back | $10 |  |
| University of Miami | 250 |  |  |  |
| **Incentives** | |  |  |  |
|  | Incentive 1 | PROTECT participants will be provided a weekly $20 incentive for completing their survey, surveillance responses and biological sample submission over a 30-week observation period. | $20 | Issue each PROTECT participant a ClinCard (reloadable debit card) that the University of Miami loads remotely as they complete project milestones. In the case of small children, the University of Miami will issue the ClinCard to the parent. |
| University of Utah | 275  For 18 months |  |  |  |
| **Incentives** | |  |  |  |
|  | Incentive 1 | Participant incentives ($100 per quarter for weekly nasal swab submissions; assume 2 quarters) | $100 | Per University policy, incentives are distributed via university approved gift cards and/or tangible gifts (participant preference). |
|  | Incentive 2 | Parent incentive ($25 per quarter per child; assume 1 child per household; assume 2 quarters) | $25 |  |
|  | Incentive 3 | Participant retention (monthly raffle: $25 gift cards; select 10 participants per month x 8 months)  10 participants per month x 18 months | $25 |  |
|  | Incentive 4 | Participant retention (PROTECT brand small gifts; enrollment and assume 2 quarters) | $10 |  |
|  | Incentive 5 | End of the study participant and parent thank you gift certificate | $100 |  |
|  | Incentive 6 | Blood draws incentive (enrollment, post-vaccine x1, end of study, assume 50% convalescent draws; assume 50 participants; 3.5 blood draws per participant)  Same assumptions with 275 total participants | $50 |  |
|  | Incentive 7 | Enrollment survey incentive | $50 |  |
|  | Incentive 8 | Vaccine card submission incentive | $50 |  |
|  | Incentive 9 | Illness survey (weekly; assume 10% get sick; assume 2 surveys) incentive  Still getting numbers on this estimate – assume all participants got sick once | $50 |  |
|  | Incentive 10 | Monthly surveys incentive  18 surveys | $25 |  |
|  | Incentive 11 | Referrals ($25 per referral; up to 4; assume 25%) | $25 |  |
